# Individualized Prediction of Breast Cancer Survival Using Flexible Parametric Survival Modeling: Analysis of a Hospital-Based National Clinical Cancer Registry

**DOI:** 10.3390/cancers13071567

**Published:** 2021-03-29

**Authors:** Donsuk Pongnikorn, Phichayut Phinyo, Jayanton Patumanond, Karnchana Daoprasert, Pachaya Phothong, Boonying Siribumrungwong

**Affiliations:** 1 Department of Clinical Epidemiology, Faculty of Medicine, Thammasat University, Pathum Thani 12120, Thailand; donsuk.p@dms.mail.go.th; 2 Cancer Registry Unit, Lampang Cancer Hospital, Lampang 52000, Thailand; karnchana.d@dms.mail.go.th; 3 Department of Family Medicine, Faculty of Medicine, Chiang Mai University, Chiang Mai 50200, Thailand; 4 Center for Clinical Epidemiology and Clinical Statistics, Faculty of Medicine, Chiang Mai University, Chiang Mai 50200, Thailand; jpatumanond@gmail.com; 5 Musculoskeletal Science and Translational Research (MSTR) Cluster, Chiang Mai University, Chiang Mai 50200, Thailand; 6 Policy and Strategy Unit, Lampang Cancer Hospital, Lampang 52000, Thailand; pachaya.p@dms.mail.go.th; 7 Division of Vascular and Endovascular Surgery, Department of Surgery, Faculty of Medicine, Thammasat University, Pathum Thani 12120, Thailand; boonying22@gmail.com; 8 Center of Excellence in Applied Epidemiology, Faculty of Medicine, Thammasat University, Pathum Thani 12120, Thailand

**Keywords:** breast neoplasms, adult, female, prognosis, statistical models

## Abstract

**Simple Summary:**

Prognostication of breast cancer patients is essential for risk communication and clinical decision-making. Many clinical tools for the survival prediction of breast cancer patients have been developed over the years. However, most of them were developed from Western countries. Studies have shown that these tools did not perform well in other ethnicities, such as Asian populations, including Thai. This study developed a new prediction model for survival predictions using modern statistical methods that allow a more accurate estimation of the baseline survival. The model was entitled the Individualized Prediction of Breast cancer Survival or the IPBS model. It contains twelve routinely available predictors that oncologists usually evaluate in daily practice. The survival information provided by the model was proven to be acceptably accurate and might be useful for physicians and patients, especially in Thailand or other Asian countries, to arrive at the most appropriate management plan.

**Abstract:**

Prognostic models for breast cancer developed from Western countries performed less accurately in the Asian population. We aimed to develop a survival prediction model for overall survival (OS) and disease-free survival (DFS) for Thai patients with breast cancer. We conducted a prognostic model research using a multicenter hospital-based cancer clinical registry from the Network of National Cancer Institutes of Thailand. All women diagnosed with breast cancer who underwent surgery between 1 January 2010 and 31 December 2011 were included in the analysis. A flexible parametric survival model was used for developing the prognostic model for OS and DFS prediction. During the study period, 2021 patients were included. Of these, 1386 patients with 590 events were available for a complete-case analysis. The newly derived individualized prediction of breast cancer survival or the IPBS model consists of twelve routinely available predictors. The C-statistics from the OS and the DFS model were 0.72 and 0.70, respectively. The model showed good calibration for the prediction of five-year OS and DFS. The IPBS model provides good performance for the prediction of OS and PFS for breast cancer patients. A further external validation study is required before clinical implementation.

## 1. Introduction

Breast cancer is the most common cancer among women and a significant public health burden worldwide [1]. Globally, the five-year prevalence of breast cancer was reported at 1.8 million cases, with an age-standardized incidence rate at 47.8 and an age-standardized mortality rate of 13.6 per 100,000 person/years [1]. In Thailand, breast cancer incidence was the highest among all other cancers, with an age-standardized incidence rate of 31.4 per 100,000 person/years, with approximately 15,000 new cases diagnosed each year [2]. The overall trends of breast cancer have been observed to be increasing in every region of Thailand. The incidence rate was projected to be around 48.5 per 100,000 person/years in 2034 [3,4,5,6]. Despite the high incidence rate of female breast cancer, the overall prognosis was favorable compared to other cancers with an estimated age-standardized five-year net survival of over 85% in developed countries [7]. In developing countries, including Thailand, the five-year net survival was somehow lower at 64.8–68.7% [7].

Cancer prognostication is essential for guiding cancer management policy at both the national and the global levels. Estimating the average prognosis from a population of individuals with specific cancer (i.e., population-based cancer registry) can provide useful information for policymakers to evaluate and benchmark the overall effectiveness of the current healthcare situation. In contrast, estimating an accurate individual prognosis for each patient with breast cancer is crucial for oncologists to arrive at the appropriate clinical management decisions due to the highly heterogeneous nature and variations of breast cancer [8]. For instance, prognostic models can provide survival predictions of patients with early breast cancer after their surgical management, allowing clinicians to determine whether adjuvant therapy will be beneficial to them.

Several survival prognostic models for patients with breast cancer were developed over the years. Some of the well-known models were as follows: the Nottingham prognostic index (NPI) [9], Adjuvant! [10], BC Nomogram [11], OPTIONS [12], CancerMath [13] and PREDICT [14]. However, all of these models were originally developed from the populations in Western countries, and some of the models were proven to provide less accurate predictions during validation in other populations, including Asian ones [15,16,17,18,19,20]. There was one study that externally validated Western prognostic models in Thailand [20]. This external validation study concluded that Western models were less accurate in Thai breast cancer patients. The poor external performance of the models could be the result of the differences in the population case-mix and regression coefficients [21]. The association and the predictive ability of the previously reported predictors in the Western models might also be different, and the re-estimation of regression coefficients is often necessary. Therefore, a prognostic model for survival prediction should be specifically developed from a dataset of Thai patients with breast cancer. This study aimed to develop and internally validate the novel prognostic models to predict the overall survival (OS) and disease-free survival (DFS) for individual patient using multicenter hospital-based clinical cancer registry data from the Network of National Cancer Institutes of Thailand.

## 2. Materials and Methods

### 2.1. Patients and Data

**Figure 1:**
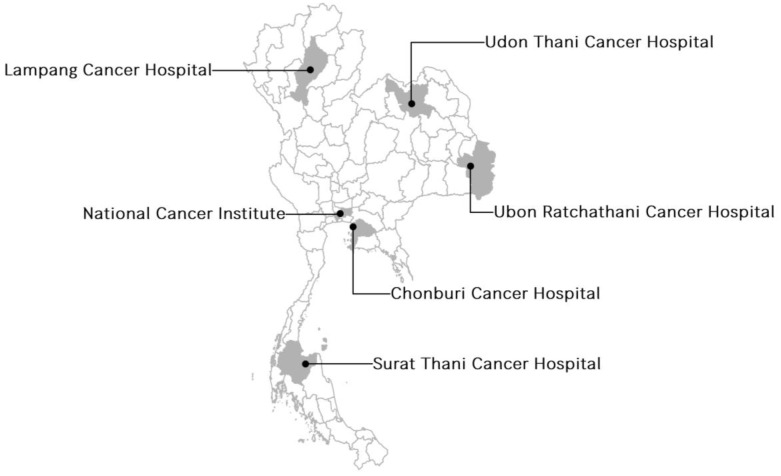
The Network of National Cancer Institute of Thailand, which contributed to the clinical database of breast cancer.

This prognostic model research was conducted based on the multicenter hospital-based clinical cancer registry data from the Network of National Cancer Institutes of Thailand, which includes the National Cancer Institute (NCI) and five regional cancer hospitals, including Lampang Cancer Hospital, Udon Thani Cancer Hospital, Ubon Ratchathani Cancer Hospital, Surat Thani Cancer Hospital and Chonburi Cancer Hospital. These cancer hospitals were located in different regions of Thailand and were responsible for approximately 10% of patients with breast cancer in Thailand (Figure 1).

The patient domain was all women newly diagnosed with invasive breast cancer who underwent surgery and were treated at the NCI and the participating regional cancer hospitals between 1st January 2010 and 31st December 2011. As our model was intended to be used for prognostication in patients with operable breast cancer patients, patients with stage IV breast cancer were excluded. We also excluded patients with any neoadjuvant therapy before surgery. Patients were followed up from the date of surgery to 31st December 2016. Overall survival (OS) was estimated from patients who died from any causes, and the definition of disease-free survival (DFS) was based on the guidelines for time-to-event endpoint definitions in breast cancer trials [22]. In our study, DFS was estimated from any invasive relapse (including ipsilateral recurrence), any appearance of a second primary cancer (including contralateral breast cancer), any appearance of distant metastasis and any causes of death, whichever occurred first and were documented in the medical records. Therefore, disease-free in our study was the length of time after surgery ends that the patient survives without any signs or symptoms or evidence of breast cancer. Patients who did not have any endpoints were censored on 31st December 2016. The study was approved by the institutional review board and ethical committee of the Faculty of Medicine, Thammasat University (MTU-EC-ES-4-211/60) and by the ethical committee of NCI and participating regional Cancer Hospitals (LPCH-EC-19/2015).

### 2.2. Predictors

Candidate predictors for the survival of patients with breast cancer were selected based on clinical importance (expert advice, previously reported prognostic models and the availability of predictors at the point of prediction). These prognostic factors included patient factors: age at diagnosis (surgery) and menopausal status; tumor factors: pathological staging, histological type, size of the tumor, histological grade, lymphovascular invasion (LVI), nodal involvement, estrogen receptor (ER), progesterone receptor (PR) and human epidermal growth factor receptor 2 (HER-2) and treatment factors: type of surgery, chemotherapy, hormonal therapy and radiotherapy.

All prognostic factor data were reviewed and retrieved from medical records. Tumor factor data were extracted directly from the pathological report. The ER or PR positivity was examined by immunohistochemistry and defined as 1% or more positive tumor cells with nuclear staining. The HER-2 positivity was defined as either a score of 2+ and 3+ by immunohistochemistry.

### 2.3. Derivation of the Survival Models

The Royston–Parmar (RP) model, a parametric alternative to the Cox’s model, was used to derive the prognostic model for overall survival and disease-free survival [23,24]. As the RP model estimates the baseline cumulative hazard function using restricted cubic splines, it provides smooth estimates of the hazard and survival functions used to extrapolate survival beyond the observed data. The number of degrees of freedom for the baseline spline function was chosen based on the lowest Akaike Information Criterion (AIC) and Bayesian Information Criterion (BIC). The proportional hazard assumption was tested graphically using Schoenfeld residuals. A multivariable fractional polynomial (MFP) procedure was used to model continuous variables, including age at surgery and size of the tumor [25]. All data analyses were performed using Stata version 16 (StataCorp, College Station, TX, USA). The derivatives of the RP model and multivariable fractional polynomial model were fitted using *stpm2* and *mfp* packages, respectively. The mathematical details of the RP model are described in Supplementary File S1.

#### 2.3.1. Missing Data

A complete case analysis would have excluded up to 30% of the original dataset due to missing values among the ten candidate predictors; multiple imputation with chained equation (MICE) was used to handle these missing values [26]. The *mi impute chained* command was used to generate missing values, and the *mi estimate* command was used to combine estimated coefficients from each RP model of the imputed datasets. We generated 20 imputed datasets during the MICE procedure based on the percentage of the variable with the highest missing values. Predictive mean matching was to impute the size of the tumor, ordinal logit models for pathological staging and histological grade and logit models for the rest of the binary predictors. The endpoint indicator and log of survival time were included in the series of chained imputation equations. The models derived from MICE were compared to the models derived from the complete-case analysis. If no significant differences were observed, the models from the complete-case analysis would be presented.

#### 2.3.2. Model Performance

The model performance was assessed in terms of discrimination and calibration. The discriminative ability assesses how well the model can distinguish between a person with more prolonged survival and shorter survival. Two measures of discrimination were selected. One is based on a concordance and another one on prognostic separation. Harrell’s C discrimination index (C-statistic) is the concordance measure that quantifies the rank between the predicted risk and the observed survival times [27]. Royston and Sauerbrei’s D statistic (D-statistic and R^2^_D_) were reported for prognostic separation measures [28]. D-statistic quantifies the observed separation between patients from low- and high-risk groups, where these two equally sized groups are dichotomized at the median value of the linear predictor of the proportional hazard model. Higher values of this statistic indicate a more remarkable discriminative ability of the model. R^2^_D_ is a measure of explained variation derived from the D-statistic.

The model calibration was evaluated with multiple measures, as follows: Firstly, the calibration plot was visualized by contrasting the observed proportion of endpoints (overall survival and disease-free survival) at a fixed time point of five years from surgery against the expected probabilities estimated from the models in 10 risk groups. To generate the ten risk categories, we estimated the linear predictors of each patient through statistical modeling and split them at the 10th, 20th, 30th, 40th, 50th, 60th, 70th, 80th and 90th centiles. Secondly, we plotted the predicted and observed survival curves in four risk groups to assess the longitudinal calibration. Cut-offs at the 25th, 50th and 75th centiles were applied to the linear predictor for generating four risk categories. Lastly, we presented the calibration slope. By regressing the observed survival outcomes on the predicted prognostic index, the regression coefficient is the calibration slope, where the values of the slope close to 1 suggest that the model is well-calibrated. Internal validity and optimism of the model were assessed by the bootstrap resampling method with 1000 replications. Optimism-corrected C-statistic, D-statistic, R^2^_D_ and calibration slope were calculated.

#### 2.3.3. Model Presentation

The hazard ratio (HR) of each predictor was presented. The regression coefficient of each predictor can be calculated by taking the natural logarithm of the hazard ratio. Baseline overall and disease-free survival probabilities (S_0_) at 5 (S_0_(5)) and 10 (S_0_(10)) years after surgery were also presented. The prognostic index (PI) can be calculated by multiplying the value of each predictor by its coefficient and then summing all the numbers together. The five-year or 10-year predicted survival probability is then calculated as S_0_(5)^exp(PI)^ or S_0_(10)^exp(PI)^. The formula of how to calculate the predicted survival probability was presented in Supplementary Table S1.

## 3. Results

### 3.1. Study Patients

**Figure 2:**
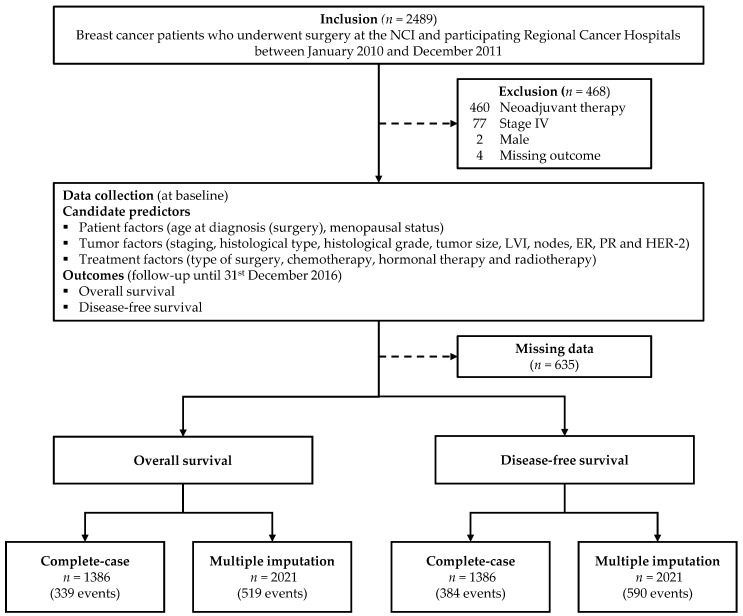
Study flow diagram of the patient cohort. NCI: National Cancer Institute. Abbreviations: NCI, National Cancer Institute of Thailand; LVI, lymphovascular invasion; ER, estrogen receptor status; PR, progesterone receptor status; HER-2, human epidermal growth factor receptor 2.

During the study period, 2489 women diagnosed with breast cancer who underwent surgical treatment were included. A total of 468 women were excluded (Figure 2). Of these patients, 519 died, 69 had local recurrence and 194 had distant metastasis before 31 December 2016. A total of 590 events were considered in the disease-free survival model. Of 2021 included cases, 1386 were available for a complete-case analysis. Regarding the complete-case analyses, the number of events was 339 for the overall survival model and 384 for the disease-free survival model. According to the reverse Kaplan–Meier method, the median follow-up time was six years for both the overall survival and the disease-free survival. The overall survival and disease-free survival probabilities at five years after surgical treatment were 77.9% and 74.0%, respectively.

Most of the patients were from the National Cancer Institute (41%). The mean age of the patients was 50 years old. A large proportion of patients had an invasive ductal tumor (95%), pathological stage II and III (85%), tumor histological grade II and III (80%), no lymphovascular invasion (60%), positive nodes (58%), positive ER and PR status (61% and 51%) and negative HER-2 status (55%). In terms of treatment, 87% of patients underwent a mastectomy, 84% received chemotherapy, 52% received hormonal therapy and 56% received radiation therapy. The demographic and clinicopathologic characteristics of the patient cohort are listed in Table 1.

### 3.2. Flexible Parametric Survival Models

The final predictors of both the overall survival and disease-free survival models are shown in Table 2. Pathological stage III, histological grade III, the presence of lymphovascular invasion and the greater number of positive lymph nodes were identified as significant predictors in the disease-free survival models. The risk was significant reduced with chemotherapy and hormonal therapy. The overall survival models also identified the statistical significance of the same set of predictors, except for pathological stage III, histological grade III and the presence of lymphovascular invasion in the complete-case analysis dataset. However, the direction and the magnitude of these nonsignificant coefficients were not different from that of the other models. Overall, the patterns of association for each predictor on the overall and disease-free survival were similar. The number of degrees of freedom for the baseline spline function that have the lowest AIC and BIC for the overall survival and disease-free survival models were degrees of freedom (df) = 2 (one interior knot) and df = 3 (two interior knots), respectively. The distributions of baseline hazard functions were shown in Supplementary Figure S1. The multiple imputation analyses gave approximately the same results. No major violations of the proportional hazard assumption were detected, and the continuous predictors (age and tumor size) were included in the models as the linear functional forms.

#### 3.2.1. Model Performance

For discrimination, the C-statistics for the complete-case and multiple imputation analyses of the overall survival models were 0.72 and 0.71, respectively. The D-statistics and R^2^_D_ were 1.246 and 0.27 for the complete-case analysis model and 1.219 and 0.26 for the multiple imputation analysis model. Since the D-statistic is an estimate of the log hazard ratio comparing two equal-sized prognostic groups, the D-statistic of 1.246 from the overall survival model can be interpreted as the risk of death in a high-risk group and is 3.48 times higher than the risk of death in a low-risk group. R^2^_D_ of 0.27 can be interpreted as 27% of the variability explained by this model on the log relative hazard scale. Regarding the disease-free survival models, the C-statistics for the complete-case and multiple imputation analysis models were 0.70 and 0.69, respectively. The D-statistics and R^2^_D_ were 1.179 and 0.25 for the complete-case analysis model and 1.129 and 0.23 for the multiple imputation analysis model.

**Figure 3:**
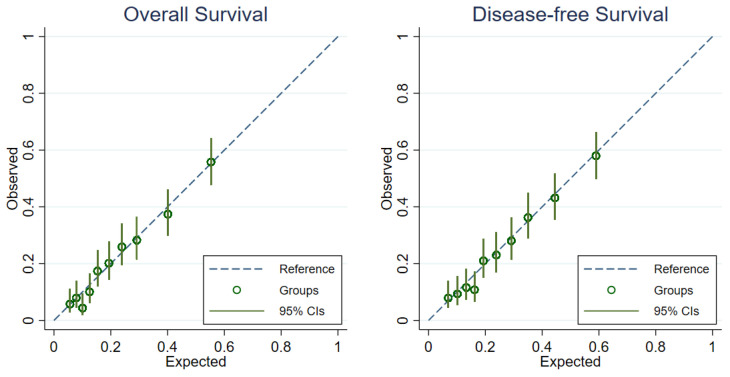
Calibration plots for predicting 5-year overall survival and disease-free survival probabilities (10 risk groups: the 10th, 20th, 30th, 40th, 50th, 60th, 70th, 80th and 90th centiles of the linear predictors).

**Figure 4:**
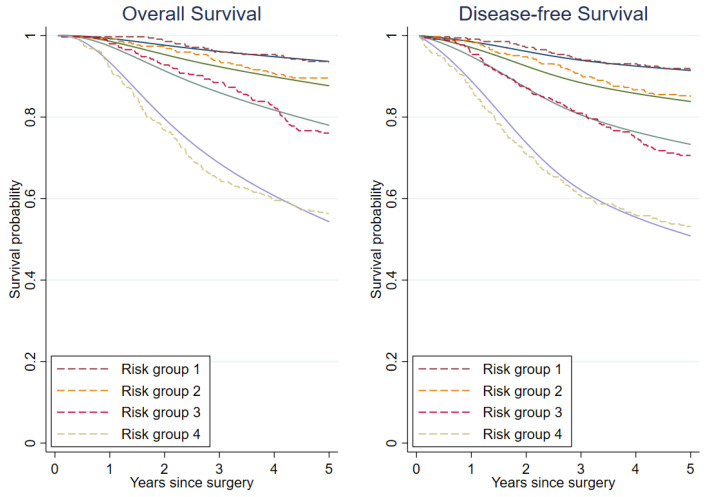
Predicted overall survival and disease-free survival curves (solid lines) compared with the observed Kaplan–Meier curves (dashed lines) (4 risk groups from the cut-offs at the 25th, 50th and 75th centiles of the linear predictors).

The overall survival and disease-free survival models appeared to be well-calibrated, according to the calibration plots comparing the predicted and the observed risks at the five-year after surgical treatment (Figure 3). Both models slightly underestimated the probability of events in the fourth risk quantile (Figure 4). The overall survival model minimally overestimated the probability of death in the second and the third risk quantiles. The disease-free survival model also slightly overestimated the probability of events in the second quantile.

For the internal validation of the complete-case analysis models, the C-statistics optimisms were 0.015 (95%CI 0.014–0.016) for the overall survival model and 0.015 (95%CI 0.014–0.016) for the disease-free survival model. The D-statistics and R_2_^D^ optimisms were 0.141 (95%CI 0.135–0.147) and 0.044 (95%CI 0.042–0.046) for the overall survival model and 0.130 (95%CI 0.124–0.136) and 0.041 (95%CI 0.039–0.043) for the disease-free survival model. The shrinkage factor for both the overall survival and disease-free survival models was 0.90.

#### 3.2.2. Model Presentation and Demonstration

For a demonstration of the model predictions, we present an example of nine patient cases with specific combinations of predictors (Table 3). The predicted five-year overall survival probabilities from the complete-case analysis were compared with the results from the multiple imputation analysis and the estimates generated by the PREDICT and NPI models. The selection of these two models was based on their ability to provide survival prediction at five years, while other models provide predictions at longer time-points that were not within our interests (i.e., 10- and 15-year survivals). The predicted survival probabilities from the complete-case analysis were comparable to the results from the multiple imputation analysis. However, the PREDICT and NPI models estimates were found to be much different from those of our models (Table 3).

**Figure 5:**
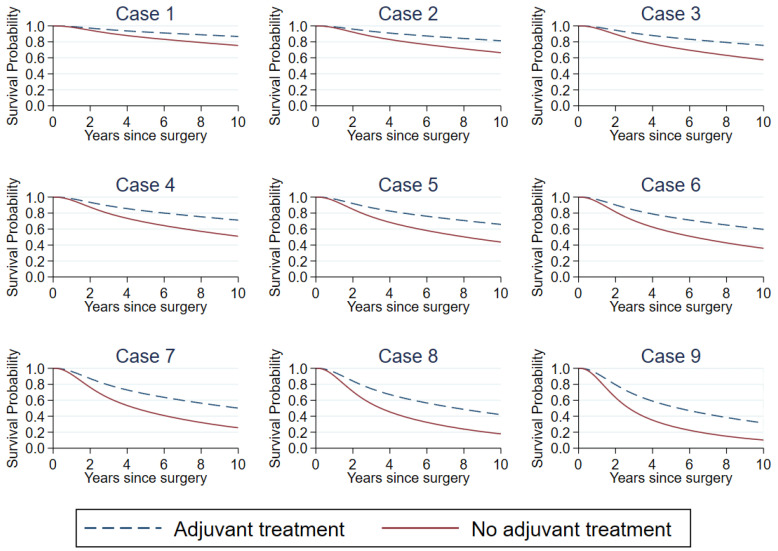
Predicted 10-year overall survival curves for nine systematically sampled patients without any adjuvant treatment after surgery and the same patients with adjuvant treatment after surgery using the complete-case analysis models.

We also demonstrated the predicted overall survival probabilities for patients without any adjuvant treatment after surgery and compared them with the predicted overall survival probabilities if those patients were prescribed adjuvant treatment after surgery (Table 4 and Figure 5). For predicting the survival probabilities without any adjuvant therapy, regression coefficients of zeros were used for adjuvant treatment predictors (0 for chemotherapy, 0 for hormonal therapy and 0 for radiotherapy). For predicting the survival probabilities if the patient received adjuvant therapy, the population proportions of patients who received each adjuvant treatment were used as the values of each predictor (0.839 for chemotherapy, 0.539 for hormonal therapy and 0.579 for radiotherapy). Then, the regression coefficients of each adjuvant treatment from the model were used for predicting the survival probabilities.

For example, the estimated five-year overall survival probability after surgery without any adjuvant treatment for a 56-year-old postmenopausal women diagnosed with 48-mm ductal breast cancer with evidence of pathological stage II, tumor histological grade III, two positive lymph nodes, lymphovascular invasion, positive ER and PR status and negative HER-2 status was 62.8%. If this patient were provided with adjuvant treatment, the five-year survival probability is projected to be increased to 79.1%.

## 4. Discussion

In this study, we derived an individual survival prediction model for Thai patients with breast cancer from a hospital-based clinical cancer registry database of the Network of National Cancer Institute of Thailand, entitled the Individualized Prediction of Breast Cancer Survival model or the IPBS model. The IPBS model is able to predict both the overall and the disease-free survival for patients with breast cancer at any time point after their surgical treatment. In making a prediction, the IPBS model requires twelve routinely available predictors in clinical practice: age at surgical treatment, menopausal status, pathological staging, tumor type, histological grading, tumor size in millimeters, lymphovascular invasion status, ER, PR and HER-2 status and type of surgical treatment. The IPBS model carries an acceptable discriminative ability, is well-calibrated and is potentially useful as a clinical tool to assist physicians in deciding whether adjuvant therapy is warranted in a given situation. Moreover, the overall and disease-free survival can help provide clinicians with a complete picture of the entire disease process.

Previous well-known prognostic models for the survival prediction of patients with breast cancer such as NPI [9], Adjuvant! [10], BC Nomogram [11], OPTIONS [12], CancerMath [13] and PREDICT [14] were developed from populations in European countries or the United States. However, it was consistently reported that patients in Asian countries, including Thailand, had significantly different clinicopathological features from Western countries. For instance, in Thailand, patients with breast cancer were considerably younger and were diagnosed with more advanced clinical staging [29,30,31]. Furthermore, Adjuvant! and PREDICT, the most well-known prognostic models, performed less accurately in patients from Asian countries, including Malaysia [16,18], South Korea [17], Taiwan [15] and Thailand [20]. The differences in the case-mix and regression coefficients both account for the drop in external performance. A difference in the population case-mix might occur because the study settings were different. For instance, when the model was developed in a secondary care center and was subsequently validated in a tertiary care setting where the distribution of predictors was often different. The regression coefficients could also be different owing to the clinical heterogeneity. When comparing the characteristics of the study patients from our model to those of PREDICT, our patients were younger (age < 50 years, 50% vs. 23%) and had a larger size of tumor (size ≥ 3 cm, 47% vs. 20%), a greater number of positive nodes (node > 4, 27% vs. 11%) and a lower proportion of them were ER-positive (61% vs. 83%). A validation study of the PREDICT model in Thailand showed that, on average, the model underestimated the five-year overall survival. A given example of the predicted five-year overall survival probabilities from nine patient cases with specific combinations of predictors demonstrated that the PREDICT and NPI models gave different predictions compared to those from our model. For example, PREDICT overestimated the five-year survival in case number 4 and case number 6 but underestimated the survival in case number 8 and case number 9. The possible explanation for this discrepancy could be the effect of the ER status, which was much higher in PREDICT. Therefore, the overestimation of survival was observed in ER-positive patients, and the underestimation was seen in ER-negative patients. Moreover, some of our significant predictors (e.g., LVI and stage) were not incorporated in PREDICT. Regarding NPI, only the tumor size, lymph node status and tumor grade were used as predictors. In addition, the predicted five-year overall survival probabilities were not for the individual patient but patients within the same prognosis category. For instance, case numbers 1, 2, 3 and 6 were in the good prognosis group, and case numbers 4, 5, 8 and 9 were in the poor prognosis group.

Many factors have been reported as the prognostic factors of patients with breast cancer [32] and have been used as predictors to predict breast cancer outcomes by several prognostic models over the years. For instance, NPI is based on the tumor size, histopathological grading and lymph node status [9]. Adjuvant! is based on the age at diagnosis, tumor size, comorbidities, histopathological grading, number of involved lymph nodes and ER [10]. PREDICT is also based on the same predictor variables as Adjuvant! but with the addition of HER-2 and KI-67 [14,33,34]. A recent systematic review of the prognostic models for breast cancer showed that the most commonly used predictors were the nodal status, tumor size, histopathological tumor grading, age at diagnosis and ER status [35]. The IPBS model includes all twelve candidate predictors selected based on the clinical importance, clinical availability and previous evidence. A full model approach was employed to avoid the data-driven selection of predictors and avoid overfitting.

Most prognostic models for breast cancer patients were developed using Cox’s proportional hazard regression [35]. However, the flexible parametric survival model or the Royston–Parmar (RP) model used in this study has many advantages over the standard Cox’s model [24], such as generating a smooth baseline hazard flexible enough to represent the true hazard function adequately. The RP model can also give a smooth survival function and extrapolate survival predictions beyond the actual follow-up time in the dataset. In this study, our RP models can predict individual smooth OS and DFS probabilities at ten years from the date of surgery. The IPBS model demonstrated good performance both in terms of model calibration and discriminative ability.

In general, an overfitting problem often arises when too many prediction parameters are incorporated into the models; however, only a small amount of optimism (the global shrinkage factor of 0.9) was identified in our study. The minimal sample size for developing the multivariable survival prediction models was generally based on three criteria, as described by Riley et al. [36]. We estimated that a minimum of 698 cases and 172 events were required for the OS model (events per predictor parameter (EPP) = 9), and a minimum of 842 cases and 233 events were required for the DFS model (EPP = 11). For the complete-case analysis models, the EPP for the OS and DFS models were 16 and 18, respectively. Thus, the study size of 1386 cases in the complete-case analysis models was adequate for the model development. Nonetheless, as missing data can lead to bias during the statistical modeling, the MICE procedure was performed [26]. As the results were comparable for both approaches (i.e., complete-case analysis and multiple imputation analysis); the regression coefficients and the baseline survival probabilities of the complete-case analysis models were reported in order to be used for the external validation study.

In this study, we modeled the treatment “drop-in” or the starting of the adjunctive treatment during the follow-up as a binary covariate. This approach is one of the seven available methods to account for treatment use after the point of prediction when developing prognostic models [37]. Although a review showed that this approach produced slightly higher-risk predictions [37], the purpose of including chemotherapy, hormonal therapy and radiotherapy in the IPBS model was not to estimate the treatment effects but to adjust for the adjuvant therapy in order to have the models capable of generating predictions of the overall survival and disease-free survival based on the prognostic factors only. We used the population proportions of patients who received each adjuvant treatment as the values of each adjuvant therapy predictors to prognosticate patients who would be given adjuvant treatment in order for physicians and patients to communicate the prognosis. It is helpful to bear in mind that the purpose of this study was not to demonstrate the treatment benefit of each adjuvant therapy but to provide useful information on whether adjuvant therapy should be initiated or not for each specific patient.

Despite the adequate study size and the rigorous statistical modeling, this study was not without limitations. Firstly, an unavoidable limitation of this study, or any prognostic model research in general, was an unavoidable time gap between data collection (i.e., during 2010–2011) and the time of model development and reporting (i.e., 2020–2021). Some novel prognostic factors might be identified during this gap and should theoretically be included in our model. For example, the data on Ki-67 [38], a specific proliferation-related gene advocated as the marker of choice for measuring and monitoring tumor proliferation, was not available when we were developing our models. In addition, the prognosis of patients with breast cancer generally improved over time as the local therapies and administration of systemic therapies has been enhanced. Secondly, the IPBS model was composed of only clinicopathologic factors. Several biological factors are associated with breast cancer prognosis, such as the urokinase plasminogen activator (uPA), plasminogen activator inhibitor (PAI-1) [39] and cathepsin D (Cath-D) [40], and were omitted, as the data were unavailable in our settings. In our future work, we will assess other potential predictive factors and their effects on the performance of our model. These will include new prognostic factors, details of treatments and comorbidity to update our prognostic models of breast cancer patients. Another limitation was that, while our prognostic models were valid for reproducibility (internal validity), our models have not yet been validated against an external, independent dataset. Since we developed our models from specialized hospital-based data, the aspect of generalizability (external validity) of our models for use in other populations of breast cancer patients in Thailand should be concerned. Therefore, where the interest lies in the model’s generalizability to other populations and settings, external validation studies are required before our models can be implemented in clinical practice. Finally, modern methods to account for the treatment used when developing the prognostic model, such as the inverse probability of treatment weighing after censoring (IPCW) or marginal structural modeling (MSM), should be used to reduce the bias in future predictions [37]. The multistate modeling of the local recurrence, distant metastasis and death may be used in the future to obtain highly personalized, dynamic predictions of the outcomes in breast cancer patients.

## 5. Conclusions

A prognostic model for the individual prediction of overall and disease-free survivals of Thai patients with early breast cancer who have undergone surgery, the IPBS model, was developed using flexible parametric survival regression. The model includes twelve routinely available predictors that were chosen based on a strong theoretical background and clinical evidence. With a good predictive ability, the IPBS model is potentially useful for providing effective risk communication and information for making an appropriate clinical decision regarding adjuvant therapy initiation for early breast cancer patients.

## Figures and Tables

**Table 1:** Demographic and clinicopathologic characteristics of the study patients.

Characteristics	Total	5-Year OS		5-Year DFS	
	*n* (%)	(%)	*p*-Value	(%)	*p*-Value
Cancer Hospital					
Lampang	343 (17.0)	77.8	0.182	67.9	0.131
Udon Thani	350 (17.3)	77.1		76.6	
Ubon Ratchathani	149 (7.4)	85.2		79.2	
Surat Thani	115 (5.7)	80.9		73.9	
Chonburi	231 (11.4)	78.0		77.5	
NCI	833 (41.2)	76.5		73.6	
Age at surgery (year, mean ± SD)	50.4 ± 10.6				
<50	1020 (50.5)	80.5	0.001	77.0	<0.001
≥50	1001 (49.5)	75.2		71.0	
Menopausal status					
Premenopause	903 (44.7)	80.2	0.012	76.6	0.005
Postmenopause	996 (49.3)	75.4		71.2	
Unknown	112 (6.0)				
Pathological stage					
I	286 (14.2)	91.3	<0.001	88.1	<0.001
II	979 (48.4)	84.9		80.8	
III	744 (36.8)	63.8		60.0	
Unknown	12 (0.6)				
Histological type					
Ductal	1915 (94.8)	77.7	0.140	73.7	0.107
Other types	106 (5.2)	82.1		79.3	
Histological grade					
I	277 (13.7)	85.2	<0.001	83.0	<0.001
II	955 (47.2)	80.0		75.5	
III	652 (32.3)	72.2		68.7	
Unknown	137 (6.8)				
Tumor size (mm, mean ± SD)	32.7 ± 20.7				
<30	1014 (50.2)	82.6	<0.001	79.1	<0.001
≥30	954 (47.2)	73.6		69.2	
Unknown	53 (2.6)				
LVI					
Yes	684 (33.9)	70.0	<0.001	65.2	<0.001
No	1205 (59.6)	82.0		79.0	
Unknown	132 (6.5)				
Node					
0	838 (41.5)	88.5	<0.001	85.7	<0.001
1–3	524 (25.9)	80.3		74.6	
≥4	659 (32.6)	62.4		58.7	
ER					
Positive	1237 (61.2)	82.7	<0.001	78.6	<0.001
Negative	718 (35.5)	69.6		66.3	
Unknown	66 (3.3)				
PR					
Positive	1026 (50.8)	84.1	<0.001	80.4	<0.001
Negative	925 (45.8)	71.1		67.2	
Unknown	70 (3.4)				
HER-2 status					
Positive	687 (34.0)	74.5	0.001	69.6	0.001
Negative	1110 (54.9)	80.6		77.7	
Unknown	224 (11.1)				
Type of surgery					
Mastectomy	1758 (87.0)	76.3	<0.001	72.6	<0.001
BCS	263 (13.0)	88.2		83.3	
Chemotherapy					
Yes	1696 (83.9)	78.1	0.685	74.2	0.645
No	325 (16.1)	76.9		72.9	
Hormonal therapy					
Yes	1053 (52.1)	85.5	<0.001	81.2	<0.001
No	900 (44.5)	69.8		66.2	
Unknown	68 (3.4)				
RT					
Yes	1126 (55.7)	75.1	<0.001	70.3	<0.001
No	819 (40.5)	82.3		79.6	
Unknown	76 (3.8)				

Abbreviations: DFS, disease-free survival; ER, estrogen receptor; HER-2, human epidermal growth factor receptor 2; LVI, lymphovascular invasion; NCI, National Cancer Institute; OS, overall survival; PR, progesterone receptor; RT, radiotherapy and BCS, breast conserving surgery.

**Table 2:** Predictors and estimated hazard ratios from multivariable flexible parametric survival models.

Predictors	OS				DFS			
	Complete-Case		Multiple Imputation		Complete-Case		Multiple Imputation	
	HR (95% CI)	*p*-Value	HR (95% CI)	*p*-Value	HR (95% CI)	*p*-Value	HR (95% CI)	*p*-Value
Age at surgery (year)	1.00 (0.99, 1.01)	0.985	1.00 (0.99, 1.01)	0.829	1.00 (0.99, 1.01)	0.784	1.00 (0.99, 1.01)	0.563
Menopausal status								
Premenopause	1		1		1		1	
Postmenopause	1.18 (0.89, 1.57)	0.241	1.17 (0.92, 1.49)	0.204	1.16 (0.89, 1.52)	0.265	1.17 (0.93, 1.46)	0.174
**Pathological stage**								
I	1		1		1		1	
II	1.04 (0.62, 1.74)	0.880	1.38 (0.91, 2.10)	0.132	1.22 (0.75, 1.99)	0.417	1.35 (0.92, 1.98)	0.126
III	1.82 (0.97, 3.41)	0.064	2.02 (1.20, 3.38)	**0.008**	1.97 (1.08, 3.58)	**0.026**	1.97 (1.22, 3.18)	**0.006**
Histological type								
Other types	1		1		1		1	
Ductal	1.49 (0.66, 3.37)	0.335	0.99 (0.63, 1.56)	0.980	1.41 (0.66, 2.99)	0.374	1.02 (0.67,1.56)	0.920
**Histological grade**								
I	1		1		1		1	
II	1.19 (0.82, 1.73)	0.360	1.25 (0.92, 1.70)	0.159	1.41 (0.98, 2.02)	0.064	1.30 (0.97, 1.74)	0.078
III	1.37 (0.93, 2.01)	0.112	1.38 (1.00, 1.89)	**0.048**	1.53 (1.05, 2.23)	**0.028**	1.38 (1.02, 1.86)	**0.038**
Tumor size (mm)	1.00 (0.99, 1.01)	0.368	1.00 (0.99, 1.01)	0.565	1.00 (0.99, 1.01)	0.458	1.00 (0.99, 1.01)	0.696
**LVI**								
No	1		1		1		1	
Yes	1.23 (0.98, 1.54)	0.072	1.38 (1.14, 1.66)	**0.001**	1.28 (1.03, 1.58)	**0.024**	1.39 (1.17, 1.65)	**<0.001**
**Node**								
0	1		1		1		1	
1–3	1.59 (1.11, 2.30)	**0.013**	1.51 (1.14, 2.00)	**0.004**	1.72 (1.23, 2.39)	**0.001**	1.60 (1.23, 2.07)	**<0.001**
≥4	2.24 (1.40, 3.59)	**0.001**	2.20 (1.51, 3.20)	**<0.001**	2.13 (1.38, 3.30)	**0.001**	1.99 (1.41, 2.82)	**<0.001**
ER								
Negative	1		1		1		1	
Positive	0.89 (0.62, 1.29)	0.545	1.04 (0.77, 1.40)	0.792	0.90 (0.64, 1.27)	0.542	1.05 (0.78, 1.40)	0.753
PR								
Negative	1		1		1		1	
Positive	0.90 (0.65, 1.25)	0.537	0.75 (0.58, 0.97)	0.028	0.96 (0.71, 1.31)	0.819	0.79 (0.62, 1.00)	0.054
HER-2 status								
Negative	1		1		1		1	
Positive	1.07 (0.86, 1.34)	0.532	1.11 (0.91, 1.35)	0.307	1.10 (0.89, 1.36)	0.363	1.10 (0.91, 1.33)	0.332
Type of surgery								
Mastectomy	1		1		1		1	
BCS	0.78 (0.52, 1.17)	0.237	0.72 (0.51, 1.02)	0.061	0.82 (0.57, 1.19)	0.295	0.79 (0.59, 1.08)	0.140
**Chemotherapy**								
No	1				1		1	
Yes	0.64 (0.45, 0.93)	**0.018**	0.63 (0.49, 0.82)	**0.001**	0.56 (0.40, 0.79)	0.001	0.62 (0.48, 0.79)	**<0.001**
**Hormonal therapy**								
No	1		1		1		1	
Yes	0.57 (0.42, 0.78)	**<0.001**	0.62 (0.48, 0.80)	**<0.001**	0.62 (0.46, 0.82)	0.001	0.63 (0.50, 0.81)	**<0.001**
RT								
No	1		1		1		1	
Yes	0.98 (0.75, 1.27)	0.857	0.89 (0.72, 1.10)	0.277	1.01 (0.78, 1.29)	0.968	0.96 (0.79, 1.18)	0.717
5-year baseline	OS = 89.3%				DFS = 88.9%			
10-year baseline	OS = 81.8%				DFS = 83.8%			

Abbreviations: CI, confidence interval; DFS, disease-free survival; ER, estrogen receptor; HER-2, human epidermal growth factor receptor 2; HR, hazard ratio; LVI, lymphovascular invasion; OS, overall survival; PR, progesterone receptor and RT, radiotherapy. Significant predictors are shown in bold.

**Table 3:** Predicted 5-year overall survival after surgery for nine systematically sampled patients using the complete-case analysis model (CC), multiple-imputation analysis model (MI), PREDICT model and Nottingham prognostic index (NPI) model.

Case	Age	Menopause	Stage	Pathology	Grade	Size	LVI	Node	ER	PR	HER-2	Surgery	5-year OS (%)			
													CC	MI	PREDICT	NPI
1	29	Pre-	1	Ductal	2	20	No	0	Pos	Pos	Neg	Mastectomy	85.4	87.2	93.0	82.0
2	54	Pre-	2	Ductal	2	40	No	0	Neg	Neg	Neg	Mastectomy	79.5	75.2	75.0	82.0
3	36	Pre-	2	Ductal	1	35	No	18	Neg	Neg	Neg	BCS	73.3	72.9	65.0	82.0
4	47	Pre-	2	Ductal	3	30	Yes	2	Pos	Pos	Neg	Mastectomy	68.5	63.9	79.0	37.0
5	56	Post-	2	Ductal	3	48	Yes	2	Pos	Pos	Neg	Mastectomy	62.8	58.2	70.0	37.0
6	51	Pre-	3	Ductal	1	50	No	8	Pos	Pos	Neg	Mastectomy	56.2	59.6	84.0	82.0
7	76	Post-	3	Ductal	2	22	No	34	Pos	Pos	Neg	Mastectomy	46.4	47.0	48.0	72.0
8	49	Pre-	3	Ductal	3	45	No	11	Neg	Neg	Neg	Mastectomy	38.1	40.3	26.0	37.0
9	33	Pre-	3	Ductal	2	150	Yes	17	Neg	Neg	Neg	Mastectomy	27.8	28.4	1.0	37.0

Abbreviations: BCS, breast-conserving surgery; CC, complete-case analysis; ER, estrogen receptor; HER-2, human epidermal growth factor receptor 2; LVI, lymphovascular invasion; MI, multiple imputation analysis; Neg, negative status; OS, overall survival; POS, positive status; PR, progesterone receptor and RT, radiotherapy.

**Table 4:** Predicted 5-year and 10-year overall survival probabilities for nine systematically sampled patients without any adjuvant treatment after surgery (No Rx) and the same patients with adjuvant treatment after surgery (Rx) using the complete-case analysis models.

Case	Age	Menopause	Stage	Pathology	Grade	Size	LVI	Node	ER	PR	HER-2	Surgery	5-year OS (%)		10-year OS (%)	
													No Rx	Rx	No Rx	Rx
1	29	Pre-	1	Ductal	2	20	No	0	Pos	Pos	Neg	Mastectomy	85.4	92.3	75.5	86.8
2	54	Pre-	2	Ductal	2	40	No	0	Neg	Neg	Neg	Mastectomy	79.5	89.1	66.5	81.4
3	36	Pre-	2	Ductal	1	35	No	18	Neg	Neg	Neg	BCS	73.3	85.5	57.5	75.6
4	47	Pre-	2	Ductal	3	30	Yes	2	Pos	Pos	Neg	Mastectomy	68.5	82.6	51.0	71.2
5	56	Post-	2	Ductal	3	48	Yes	2	Pos	Pos	Neg	Mastectomy	62.8	79.1	43.7	65.8
6	51	Pre-	3	Ductal	1	50	No	8	Pos	Pos	Neg	Mastectomy	56.2	74.8	35.9	59.6
7	76	Post-	3	Ductal	2	22	No	34	Pos	Pos	Neg	Mastectomy	46.4	67.9	25.5	50.2
8	49	Pre-	3	Ductal	3	45	No	11	Neg	Neg	Neg	Mastectomy	38.1	61.4	17.9	42.0
9	33	Pre-	3	Ductal	2	150	Yes	17	Neg	Neg	Neg	Mastectomy	27.8	52.4	10.2	31.6

Abbreviations: BCS, breast-conserving surgery; CC, complete-case analysis; ER, estrogen receptor; HER-2, human epidermal growth factor receptor 2; LVI, lymphovascular invasion; MI, multiple imputation analysis; Neg, negative status; OS, overall survival; POS, positive status; PR, progesterone receptor; RT, radiotherapy and Rx, adjuvant treatment after surgery.

## Data Availability

The datasets used and/or analyzed during the current study are available from the corresponding author upon reasonable request.

## Supplementary Materials

The following are available online at https://www.mdpi.com/article/10.3390/cancers13071567/s1: Table S1: Predictors, scoring and formulas for calculating the overall and disease-free survival probabilities, File S1: Royston–Parma model, Figure S1: Baseline hazard rates of the overall survival and disease-free survival models estimated from different survival models.
