# Supplementary material for: Individualized Prediction of Breast Cancer Survival Using Flexible Parametric Survival Modeling: Analysis of a Hospital-Based National Clinical Cancer Registry
Source: Cancers (Basel). 2021 Mar 29;13(7):1567. doi: 10.3390/cancers13071567 (PMC8037061; doi:10.3390/cancers13071567)
Supplement: Supplementary file 1 [file cancers-13-01567-s001.pdf]

cancers

Supplementary Materials:

Individualized Prediction of Breast Cancer Survival Using Flexible Parametric Survival Modeling: Analysis of a Hospital-Based National Clinical Cancer Registry

Donsuk Pongnikorn, Phichayut Phinyo, Jayanton Patumanond, Karnchana Daoprasert, Pachaya Phothong and Boonying Siribumrungwong

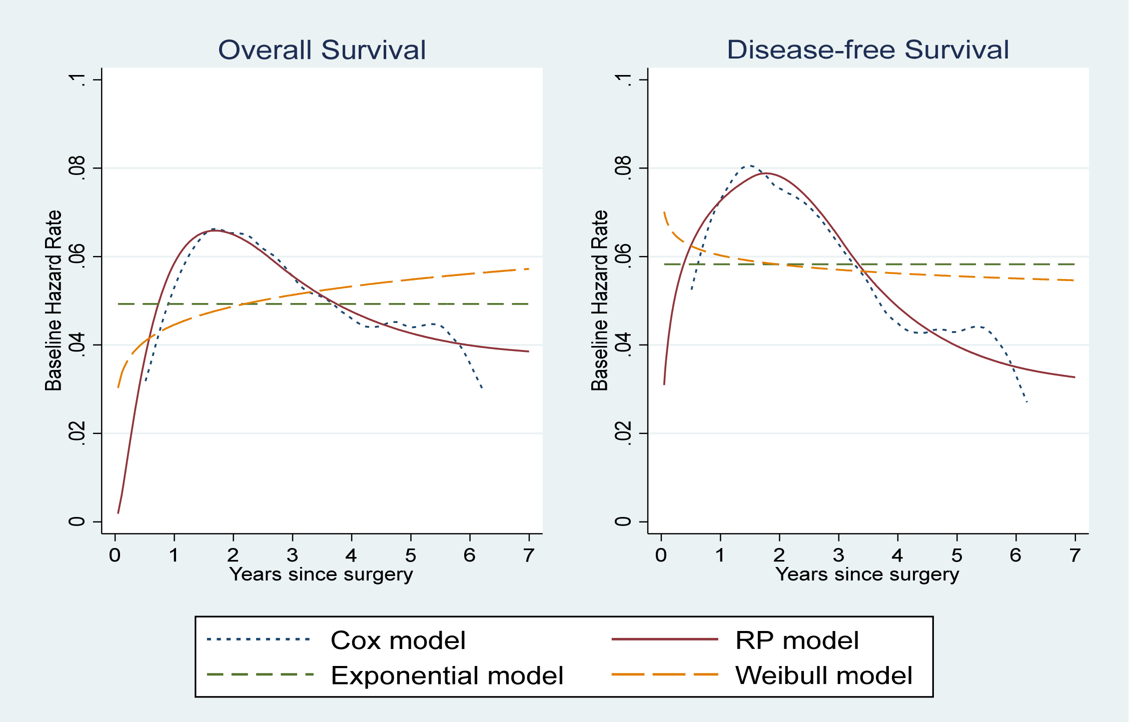

Figure S1. Baseline hazard rates of overall survival and disease-free survival models estimated from different survival models.

Table S1. Predictors, scores, and formulas for calculating predicted survival probabilities.

Predictors		Score
AGE	Age at surgery (year)	Age at surgery - 50
MENO	Menopausal status	0 = Pre-menopause,
		1 = Post-menopause
SURG	Type of surgery	0 = Mastectomy,
		1 = Breast conserving surgery
STG	Pathological stage	
	I	STG2 = 0, STG3 = 0
	II	STG2 = 1, STG3 = 0
	III	STG2 = 0, STG3 = 1
HIST	Histological type	0 = Others, 1 = Ductal
GRD	Histological grade	
	I	GRD2 = 0, GRD3 = 0
	II	GRD2 = 1, GRD3 = 0
	III	GRD2 = 0, GRD3 = 1
SIZE	Tumor size (mm)	Tumor size - 30
LVI	Lympho-vascular invasion	0 = No, 1 = Yes
NODE	Number of positive axillary lymph nodes (node)	
	0	NODE2 = 0, NODE3 = 0
	1-3	NODE2 = 1, NODE3 = 0
	≥4	NODE2 = 0, NODE3 = 1
ER	Estrogen receptor status	0 = Negative, 1 = Positive
PR	Progesterone receptor status	0 = Negative, 1 = Positive
HER2	HER-2 status	0 = Negative, 1 = Positive
CHEM	Chemotherapy	0 = no adjuvant therapy
		0.839 = received adjuvant therapy
HORM	Hormonal therapy	0 = no adjuvant therapy
		0.539 = received adjuvant therapy
RADI	Radiotherapy	0 = no adjuvant therapy
		0.579 = received adjuvant therapy
OS_5	Baseline 5-year overall survival probability	0.893
OS_{10}	Baseline 10-year overall survival probability	0.818
DFS_5	Baseline 5-year disease-free survival probability	0.889
DFS_{10}	Baseline 10-year disease-free survival probability	0.838
PI_{OS}	Prognostic index of overall survival	0.0001*AGE + 0.1681*MENO - 0.2428*SURG
		+ 0.0398*STG2 + 0.5962*STG3 + 0.4004*HIST + 0.0021*SIZE
		+ 0.4655*NODE2 + 0.8066*NODE3 + 0.2071*LVI
		+ 0.1737*GRD2 + 0.3126*GRD3 - 0.1122*ER - 0.1037*PR
		+ 0.0714*HER2 - 0.4421*CHEM - 0.5539*HORM - 0.0246*RADI
PI_{DFS}	Prognostic index of disease-free survival	0.0018*AGE + 0.1510*MENO - 0.1956*SURG
		+ 0.2018*STG2 + 0.6774*STG3 + 0.3416*HIST + 0.0016*SIZE
		+ 0.5409*NODE2 + 0.7573*NODE3 + 0.2435*LVI
		+ 0.3422*GRD2 + 0.4226*GRD3 - 0.1073*ER - 0.0358*PR
		+ 0.0973*HER2 - 0.5737*CHEM - 0.4825*HORM + 0.0050*RADI
Predicted 5-year overall survival probability		$OS_5^{\exp(PI_{OS})}$
Predicted 10-year overall survival probability		$OS_{10}^{\exp(PI_{OS})}$
Predicted 5-year disease-free survival probability		$DFS_5^{\exp(PI_{DFS})}$
Predicted 10-year disease-free survival probability		$DFS_{10}^{\exp(PI_{OS})}$

Supplementary File S1: Royston-Parmar model [1–3]

When regression modeling is used for prognostic models, Cox proportional hazard model is a common regression model type because of its ease of calculating the relative effects of hazards between groups (i.e., hazard ratio (HR)) without needing to estimate the baseline hazard function. Therefore, the only measures that can be estimated at the event times are absolute measures of effects (e.g., survival probability, hazard rates), which results in a step function where the estimate at one event is held constant and carried forward until the time of the next event. Moreover, the important assumption is proportional hazards where any two hazard rates predict by the model are proportional over time. In other word, we assume that relative hazard (i.e., HR) is constant over time. Cox proportional hazard model can be algebraically written as:

$$h_i(t \mid x_i) = h_0(t)\exp(\beta x_i) \quad (1)$$

$h_i(t \mid x_i)$ is the hazard function of the ith individual and conditional on covariates x_i and the baseline hazard function $h_0(t)$ is $h_i(t \mid x = 0)$ when all the covariates x_i equals to zero. $\beta = \beta_1, \ldots, \beta_k$ is the vector of the regression coefficients. The appealing feature of the Cox proportional hazard model is that we do not need to assume that the baseline hazard function has a specific shape.

Since the optimal approach for prognostic models would be to utilize the baseline hazard function for the continuous mathematical estimation of the absolute measures of effect, by ignoring the baseline hazard, we are missing out some useful information such as the absolute risk (i.e., difference in survival or mortality rates). Furthermore, overfitting problem can arise because of the lack of baseline hazard specification. It means that the model is too closely matched to the data that it is based on and may not generalize well to other scenarios.

Parametric survival models (or parametric proportional hazard model) permit direct estimation of absolute measures of effect because an underlying distribution is specified mathematically and allows extrapolation of survival estimates outside the study observation time and this is not possible with a Cox proportional hazard model. Parametric survival models specify the baseline hazard ($h_0(t)$), and a common specification is the Weibull distribution, which is a function of a scale parameter (λ), a shape parameter (γ), and time (t), the baseline hazard function of the Weibull model is written as:

$$h_0(t) = \lambda\gamma t^{\gamma-1} \quad (2)$$

The shape of the Weibull function depends on the shape parameter (γ), which generally is a monotonically increasing ($\gamma > 1$) or decreasing ($\gamma < 1$) hazard; and when the shape parameter equals one ($\gamma = 1$), the baseline hazard is reduced to an exponential distribution with a constant hazard. However, "real-life" data may show that hazards peak at certain points in time followed by a decline, parametric survival models like the exponential and Weibull models are not flexible enough to capture the shape of the baseline hazard function.

Royston-Parmar model (RP model) [19], a type of flexible parametric survival model, solves issues encountered when using the cox proportional hazard and parametric proportional hazard models. Royston-Parmar model features a restricted cubic spline. Cubic splines are polynomial segments ($\beta_0 + \beta_1 x + \beta_2 x^2 + \beta_3 x^3$) that smoothly join at intervals called "knots". At the knot locations, cubic functions are forced to join resulting in a continuous function with flexibility incorporated. A restricted cubic spline is a cubic spline with an additional restriction where the first and last sub-functions beyond the boundary knots are linear functions instead of cubic functions. Since the RP model can fit of the restricted cubic spline on many scales (i.e., the proportional hazard (PH) scale, the proportional odds (PO) scale, or the probit scale), in this study, we use the flexible parametric proportional hazard model which is an extension of the Weibull model. In the PH scale, the Royston-Parmar model can be thought of as a generalization of the Weibull distribution where a general function for the baseline log cumulative hazard function on the log timescale is modeled instead of a linear function.(as is the case when a Weibull distribution is pre-specified). The log cumulative baseline hazard function on the log timescale for a Weibull distribution is written as:

$\ln(H_0(t)) = \ln\lambda + \gamma_1\ln(t) = \gamma_0 + \gamma_1\ln(t)$ (3)
where $\ln(H_0(t))$ is linear function of $\ln(t)$

The above can be extended by including a restricted cubic spline function of $\ln(t)$:
$\ln(H_0(t)) = \gamma_0 + \gamma_1\ln(t) + \gamma_2 z_1\ln(t) + \gamma 3 z_2\ln(t) + \ldots$ (4)

$\ln(t)$, $z_1\ln(t)$ and $z_2\ln(t)$ are the basis functions of the restricted cubic spline. The number of knots or degrees of freedom specifies the number of basis functions. The derivation of these basis functions is described below.

We first define m interior knots ($k_1, \ldots, k_m$) in addition to the two boundary knots, k_{min} and k_{max} which are placed at the minimum and maximum of $\ln(t)$ respectively. m interior knots have m+1 degrees of freedom and m+1 basis functions.

Equation 4 can also be rewritten as:
$s(\ln(t) \mid \gamma) = \gamma_0 + \gamma_1 z_1 + \gamma_2 z_2 + \gamma_3 z_3 + \ldots + \gamma_{m+1} z_{m+1}$ (5)

z_j variables are derived as follows:
$z_1 = \ln(t)$ (6)
$z_j = (\ln(t) - k_j)_+^3 - \lambda_j(\ln(t) - k_{min})_+^3 - (1 - \lambda_j)(\ln(t) - k_{max})_+^3$ (7)
the "+" notation denotes $(x)_+ = \max(0, x)$

where for j=2, …, m+1,
$\lambda_j = \frac{k_{max} - k_j}{k_{max} - k_{min}}$ (8)

For instance, a baseline spline function with two interior knots (in addition of two boundary knots) or three degree of freedom (df(3)) can be written as,
$s(\ln(t) \mid \gamma) = \gamma_0 + \gamma_1 z_1 + \gamma_2 z_2 + \gamma_3 z_3$ (9)

One interior knot or two degree of freedom (df(2)): $s(\ln(t) \mid \gamma) = \gamma_0 + \gamma_1 z_1 + \gamma_2 z_2$ (10)

No interior knot or one degree of freedom (df(1)) or Weibull model: $s(\ln(t) \mid \gamma) = \gamma_0 + \gamma_1 z_1$ (11)

The Royston-Parmar model under a PH context can be expressed mathematically as:
$\ln(H(t) \mid x_i) = s(\ln(t) \mid \gamma) + \beta x_i$ (12)

Survival function, $S(t)$, at time t for an individual subject can then be defined as:
$S(t) = S_0(t)^{\exp(\eta)}$ (13)
where $S_0(t)$ is the baseline survival function = $\exp(-\exp(s(\ln(t) \mid \gamma)))$ and η is the linear predictor of the model.

Reference

1. Royston, P.; Lambert, P.C. *Flexible Parametric Survival Analysis Using Stata: Beyond the Cox Model*. 1st edition. Stata Press: College Station, TX, USA, 2011, 339 p.
2. Ng, R.; Kornas, K.; Sutradhar, R.; Wodchis, W.P.; Rosella, L.C. The current application of the Royston-Parmar model for prognostic modeling in health research: A scoping review. *Diagn. Progn. Res.* **2018**, *2*, 4.
3. Berhane, S.; Fox, R.; García-Fiñana, M.; Cucchetti, A.; Johnson, P. Using prognostic and predictive clinical features to make personalised survival prediction in advanced hepatocellular carcinoma patients undergoing sorafenib treatment. *Br. J. Cancer.* **2019**, *121*, 117–124.
